# Characteristics of Piglets Born by Two Highly Prolific Sow Hybrids

**DOI:** 10.3389/fvets.2020.00355

**Published:** 2020-06-24

**Authors:** Sarah-Lina Aagaard Schild, Leslie Foldager, Lena Rangstrup-Christensen, Lene Juul Pedersen

**Affiliations:** ^1^Department of Biosystems and Technology, Swedish University of Agricultural Sciences, Alnarp, Sweden; ^2^Department of Animal Science, Aarhus University, Tjele, Denmark; ^3^Bioinformatics Research Centre, Aarhus University, Aarhus, Denmark

**Keywords:** piglet mortality, organic pig production, piglet viability, lactating sows, outdoor farrowing, animal welfare, sow genetics, sow parity

## Abstract

High piglet mortality constitutes a welfare challenge in Danish organic pig production with almost one in three piglets dying before weaning. Piglet characteristics such as birth weight, rectal temperature and intrauterine growth restriction (**IUGR**) affect piglet survival. Due to differences in breeding goals, these characteristics may be expected to differ between sow hybrids. Thus, the aims of the present study were (1) to investigate piglet characteristics in two highly prolific sow hybrids and (2) to study to which extent the aforementioned characteristics affect piglet mortality. Forty-nine sows (22 DanBred and 27 Topigs Norsvin) were followed in their first two parities. Sows were housed outdoors and gave birth in huts. On day 1 *postpartum* (***pp***) piglets were individually marked, weighed, their rectal temperature was recorded and they were scored for IUGR. Weight and rectal temperature were recorded again 3 days *pp*. Principal component analyses were conducted to explore relationships among variables. Early piglet death grouped with IUGR, lower rectal temperature and weight on day 1 *pp*. Late mortality grouped with increasing litter size and DanBred hybrid. Whilst, Topigs Norsvin hybrid grouped with increasing rectal temperature day 3 *pp*, longer crown to rump length, higher weight and more teats on the sow. Results of the statistical analyses showed that Topigs Norsvin piglets were heavier 1 and 3 days *pp* (*p* < 0.001) compared to DanBred piglets. Furthermore, Topigs Norsvin piglets had a higher rectal temperature than DanBred on day 1 *pp* (*p* = 0.023). The risk of IUGR depended on an interaction between sow hybrid and parity (*p* = 0.023). DanBred sows gave birth to more piglets (18.2 ± 0.6) than Topigs Norsvin sows (15.7 ± 0.5, *p* = 0.003), however, DanBred sows had fewer teats than Topigs Norsvin sows. Weight on day 1 *pp* affected both the odds of stillbirth (*p* < 0.001) and live born death (*p* < 0.001). Lower rectal temperature day 1 *pp* (*p* < 0.001) increased the odds of live born death. In conclusion, the investigated hybrids differed in several piglet characteristics related to piglet mortality. Use of sows giving birth to heavier and fewer piglets in the litter may thus be a useful tool to reduce piglet mortality in pig production with outdoor farrowing.

## Introduction

At present, a major challenge in pig production with outdoor farrowing (organic and free-range) is high piglet mortality. In Europe the piglet mortality in organic production is higher than in conventional indoor systems (1, 2) and a recent study showed that close to one in three piglets die before weaning, at 7 weeks of age, in Danish organic pig production (3). Characteristics lowering survival chances of piglets in conventional indoor and in outdoor production comprise e.g.: low birth weight (4, 5), low rectal temperature in the first 24 h *postpartum (****pp****)* (4, 5) and increased birth litter size (4, 6). Thus, one way to improve piglet survival in organic production would be to change the aforementioned litter/piglet characteristics through choice of sow genetics. In systems with outdoor farrowing, management tools, which are commonly used in indoor production (e.g., split suckling, birth surveillance and assistance, use of milk replacer and colostrum supplement) are difficult to apply. This further emphasizes the need for production of viable, and preferably self-sustaining, piglets in such systems. Additionally, large litter sizes, even when consisting of viable piglets, pose a challenge in outdoor systems due to the extensive housing conditions. Indoors, a common practice used to handle surplus piglets (piglets for which there are no available teats on the sow) is the use of nurse sows. Under outdoor conditions, it is possible (although difficult) to create nurse sows. In practice some farmers do choose to use nurse sows, others leave the large litters with the sow and let “nature run its course” and other farmers again have established a routine where they systematically euthanize surplus piglets starting with the least viable. None of these routines are efficient, the latter two are morally questionable and conflict with the ethical principles of organic farming put forward by IFOAM (The International Federation of Organic Agriculture Movements). The use of nurse sows can be argued to challenge the principle of ecology (7) and the concept of naturalness of the system with regards to the agro-ecological approach (8). According to this approach, the farm “*should be transformed into a complex, sustainable, and balanced agro-ecosystem”* (8). Thus, in a system complying with the agro-ecological approach a sow would not give birth to more piglets, than she is able to nurse and which do not have a reasonable chance of survival.

There is a need to study piglet survival when using lower prolific sow hybrids, or breeds, in outdoor systems. However, in Europe, the sows used for organic pig production are often the same highly prolific hybrids as those used in conventional indoor production (9). Therefore, these hybrids were the focus of the present study. In Denmark, commonly the DanBred Landrace x Yorkshire hybrid sows are used. These sows are, among other traits, bred for a high number of living pigs in the litter 5 days *pp* (10) and the Danish national average litter size for this hybrid was reported to be 19.0 total born piglets in 2018 (11). The Topigs Norsvin F1 hybrid TN70 (Landrace x Z-line) sow on the other hand is, among other traits, bred for the sow being able to nurse her own litter [“*Every extra piglet born should be nursed and weaned by its own mother”* (12)]. Such different selection parameters likely affect both the birth litter size and the characteristics, and hence the viability, of the piglets born. Therefore, the aims of the present study were (1) to investigate the piglet characteristics in two highly prolific sow hybrids (DanBred and Topigs Norsvin TN70) under free-range conditions and (2) to investigate if the piglet characteristics affected piglet mortality. Based on the selection criteria for the two sow hybrids it was expected that Topigs Norsvin sows would give birth to fewer, heavier piglets with a higher rectal temperature compared to DanBred sows. Furthermore, lower weight and rectal temperature in early lactation and signs of intrauterine growth restriction (**IUGR**) were expected to increase the risk of stillbirth and of live born piglets dying.

## Materials and Methods

### Animals

This study was conducted at the experimental free-range farm at the Department of Animal Science, Aarhus University from November 2016 to October 2017. The sows included in the study were DanBred Hybrid F1-crossed sows (**DanBred**) based on DanBred Landrace and DanBred Yorkshire and Topigs Norsvin F1 hybrid (**TN70**) crossbred sows from Norsvin Landrace and Topigs Norsvin Z-line (Yorkshire). The sows were purchased as gilts from one multiplier herd in Denmark (gilts were selected from ~23 different litters) and one multiplier herd in Norway (gilts represented animals from ~10 different litters), respectively. They arrived in one batch to the experimental farm at an age of 14–22 weeks. All sows were artificially inseminated for first parity in their second oestrus (22 weeks of age, average body weight 197 ± 3 kg) to form two batches with ~9 weeks in between expected farrowing date. They were inseminated with semen from 6 known DanBred Duroc boars, where semen from each boar was used in a balanced design on both sow hybrids. For second parity, the sows were serviced in their first oestrus after weaning. A total of 49 animals (22 DanBred and 27 TN70) gave birth to 88 litters during the study period. Sows were followed through their first two parities. Not all sows gave birth in both parities, 8 sows only gave birth in first parity, 2 only in second parity and the remaining 39 sows gave birth in both parities. The reasons for sows not giving birth in both parities were that some sows had a late oestrus, some did not become pregnant and one sow was injured and had to be euthanized in first parity. The sows gave birth in four batches; batch “1” autumn, “2” winter, “3” spring and “4” summer. Batch 1 and 2 represented sows in their first parity and batch 3 and 4 represented the same sows in their second parity.

#### Gestation Housing

Prior to parturition, the sows were housed in groups of 10–14 in the gestation field. The paddocks in the gestation field measured 40 × 100 m and in each paddock sows had access to two communal huts (L:600 cm, W_top_:110 cm, W_bottom_:300 cm, H:110 cm). As part of another experiment, sows were grouped—balanced across hybrids—according to protein level in their diet throughout the two gestation periods. Sows were fed either a normal (NE 7315 kJ/kg, crude protein 11.1%; Green So Drægtig, Vestjyllands Andel A.m.b.a., Ringkjøbing, Denmark) or low (NE 7338 kJ/kg, crude protein 10.2%; Vestjyllands Andel A.m.b.a., Ringkjøbing, Denmark) crude protein level in their diet. Sows were fed ~3.8 kg/sow/day from service until day 60 in pregnancy, they were fed in individual feeding stalls. From day 60–100 in gestation they received ~4.7 kg/sow/day and until parturition ~5 kg/sow/day. Sows in all paddocks had *ad libitum* access to water (in a trough positioned ~20 m away from the hut) and roughage (consisting of clover grass silage in batch 1 and 2 and the paddock grass cover in batch 3 and 4).

#### Farrowing and Lactation Housing

Ten days before expected parturition of the first sow in each batch, sows were moved to individual paddocks (18 × 25 m) in the farrowing field. In their paddock, sows had access to a farrowing hut. Twelve sows per batch had access to a communal hut [Center of Development for Outdoor Livestock Production, Marsvej 43, DK-8960 Randers, Denmark; see Schild et al. (13) for a detailed description] while the other half of the sows had access to an insulated A-frame hut. Sows were randomly distributed to hut design within sow hybrid and protein level in the diet.

The communal hut housed four sows individually (each compartment measured L:240 cm, W:250 cm), but under the same roof. In each of the four compartments piglets had access to a covered and heated (Eheater, Orbital A/S, Trykkerivej 5, 6900 Skjern, Denmark) piglet creep area, with an insulating rubber mat (AAG Aalborg Gummivarefabrik A/S, Sundsholmen 3, 9400 Nørresundby, Denmark) placed in the bottom of the creep (not present in batch 1). The A-frame huts measured L:230 cm, W_top_:110 cm, W_bottom_:190 cm, H:110 cm). Both Communal and A-frame huts were provided with plenty of barley straw (~7.6 kg/m^2^ in summer, 10 kg/m^2^ during spring and autumn and 12.6 kg/m^2^ during winter) prior to sow relocation to the farrowing field. After parturition, additional bedding was provided as needed, the need was evaluated by the animal caretakers.

Prior to parturition, a roller (16 cm high, communal huts) or board (15 cm high, A-frame huts) was placed in the sow entrance to each farrowing hut and left until 10 days *pp*. The roller/board prevented piglets from leaving the huts, whereas sows were still able to leave the huts to roam freely within their individual paddocks. Furthermore, during autumn and winter, plastic curtains were placed in the sow entrance of each hut to lower drafts. During batch 3 and 4 (spring and summer), wallows were created in all paddocks to allow sows to wallow and control their thermoregulation. No birth assistance was provided. Male pigs were surgically castrated 3 days after birth using analgesia (0.04 to 0.05 mL Flunixin 50 mg) but no teeth resection, tail docking or iron injection were done.

At relocation to the farrowing field, each sow received ~4 kg of feed daily, in the morning. Feeding level was then gradually increased from parturition and until weaning where the level was at ~11 kg/sow/day. Sows allocated to normal protein level during gestation also received normal protein level during lactation (NE 7623 kJ/kg, crude protein 14.4%) while sows allocated to low protein level during gestation also received low protein level during lactation [NE 7554 kJ/kg, crude protein 12.5% (Vestjyllands Andel A.m.b.a., Ringkjøbing, Denmark)]. Sows had *ad libitum* access to water (in a trough) and roughage (batch 1 and 2 clover grass silage; batch 3 and 4 clover grass cover in the paddock).

Between farrowing batches huts were relocated to new paddocks that had been without sows for at least 5 months.

#### Recordings

Within the first week after relocation to the farrowing paddocks, the number of functional teats on each sow was visually assessed and recorded.

On day 1 *pp* (defined as between 24 and 48 h after birth of the first piglet) all piglets were counted, inspected, individually weighed and ear tagged. Their rectal temperature (recorded with a Kruuse Digi-Temp Express, Jørgen Kruuse A/S, Havretoften 4, 5550 Langeskov, Denmark) and gender were recorded, crown to rump length (**CTR**) was measured and the piglets were scored for signs of IUGR. Presence of IUGR was scored to piglets displaying all the three following physical characteristics: a steep and dolphin-like forehead, bulging eyes and perpendicular wrinkles on the snout [as described by Hales et al. (14)] and the score “no presence” of IUGR was given to piglets not showing all the aforementioned characteristics. Piglets weighing <700 g were considered non-viable {piglets with a birth weight <1 kg have low viability [e.g., Quiniou et al. (15), Rangstrup-Christensen et al. (3)]} and euthanized by blunt force trauma.

At day 3 *pp*, piglets were once again counted, inspected, weighed and their CTR and rectal temperature were recorded.

In cases where sows gave birth to a surplus of piglets (more piglets than the sow had functional teats), cross fostering was done within hybrid once between 1, defined as 24 h after the birth of the first piglet, and 3 days of parturition. Piglets were always added to an evenly aged or younger litter than they were taken from. In cases where cross fostering was not possible (no sows with spare teats) the smallest of the surplus piglets were euthanized for ethical reasons by blunt force trauma.

During the study, humane endpoints were defined as: sow/piglet unable to stand on own accord, sow/piglet has severe injury (broken bone, deep wound), piglet is emaciated (conspicuous ribs, backbone and hipbones).

#### Piglet Necropsy

All piglets that died in the farrowing field were collected for necropsy. At collection, the date and piglet ear tag number were recorded. It was also noted whether each piglet was euthanized or if it had died of its own accord. After collection, dead piglets were stored in a freezer until 24–36 h before necropsy where the piglets were thawed at room temperature. At necropsy, the ultimate cause of death of each piglet was identified [as described by Rangstrup-Christensen et al. (3)]. Piglets were categorized as being “stillborn” if their lung tissue sank when suspended in water. Piglets with subcutaneous oedema, internal and/or external lacerations and/or fractures were scored as having died from “crushing.” “Euthanized” piglets were identified from the note made during collection and piglets, where the cause of death could not be established during necropsy, were scored, as cause of death is “unknown”. “Other” referred to piglets diagnosed with a cause of death other than stillborn, crushed or euthanized.

#### Statistical Analyses

Exploratory principal component analyses (**PCA**) were carried out using the FactoMineR package (16) in R version 3.6.3 (17). All other Statistical analyses were conducted in SAS software (SAS 9.4, SAS Institute Inc., Cary, NC, USA) and with a significance level of 5%.

##### Piglet characteristic in two sow hybrids

For analyses applying piglets as experimental unit, covariance between piglets from the same litter was accounted for by including litter as a random effect. Likewise, covariance between litters from the same sow was accounted for by including sow as a random effect whenever possible. The random effect of sow being relevant also for models considering litter as the experimental unit. In case of non-convergence, the random effect of sow was removed from the model. The influence of the boar was adjusted for in models using piglets as experimental unit by including this as a nuisance fixed effect, which was kept but not considered further in results. The fixed effects of main interest were hybrid (DanBred/TN70) and parity (first/second) and results for these are reported, significant or not. Hut design (A-frame/communal) and protein (normal/low) were included as fixed effects and always kept but results for these are only given explicitly when statistically significant. In addition, interaction between hybrid and parity, litter size and teat number were included at outset but removed from the final model if statistically non-significant. Teat number was not significant in any of these analyses.

*Weight and rectal temperature.* The response variables piglet weight at first inspection (day 1 *pp*) and at castration (day 3 *pp*) as well as piglet's average daily weigh gain till weaning (in week 6 *pp* or earlier) were all analyzed using the following final linear mixed effects model.

(Model 1) $$\begin{array}{l} Y_{ijkl}=\beta_{0}+\beta_{1}H_{TN70}+\beta_{2}P_{second}+\beta_{3}A_{hut}+\beta_{4}F_{low}+\beta_{5}L_{size}\\ \;+B_{duroc}\;+\gamma_{j\left(i\right)}+\delta_{k\left(j\right)}+\varepsilon_{ijkl} \end{array}$$

Here β_1_ is the parameter describing the effect of sow hybrid with *H*_*TN*70_ being an indicator variable (0 for DanBred and 1 for TN70) and β_2_ is the corresponding parameter for sow parity with *P*_*second*_ an indicator, which is 0 for first parity and 1 for second parity. For brevity, we have omitted indices from these indicator variables, but the indices on *Y* and elsewhere in the model correspond to piglet *l* in litter (and parity) *k* for sow *j* of hybrid type *i*. For simplicity, the boar effect has briefly been denoted *B*_*duroc*_ and can be expressed by either five indicator variables with parameters β_6_ to β_10_, or by a six leveled categorical variable with one reference level included in the intercept parameter β_0_ and corresponding changes of index letters to include the counting of the six boars. The other fixed effect variables of the model are hut design (*A*_*hut*_ : 0 for communal and 1 for A-frame), protein level in feed (*F*_*low*_ : 0 for normal and 1 for low) and litter size (*L*_*size*_) with corresponding parameters β_3_, β_4_ and β_5_, respectively. Finally, normal distributed random effects of sow and litter are denoted by γ and δ, and ε is the normal distributed residual error. However, the random effect of sow could not be estimated in the model for average daily weight gain and instead, a compound symmetry residual correlation structure for observations from same sow was included.

For the analyses of rectal temperature at first inspection and at castration, litter averages were used as response to accommodate left skewness. Model 1 was therefore adjusted by omission of the boar effect and the random effect δ for litter and the letter *l* indexing piglets.

(Model 2) $$\begin{array}{l} Y_{ijk}=\beta_{0}+\beta_{1}H_{TN70}\;+\beta_{2}P_{second}+\beta_{3}A_{hut}+\beta_{4}F_{low}\;+\beta_{5}L_{size}\\ \;+\gamma_{j\left(i\right)}+\varepsilon_{ijk} \end{array}$$

Results of the analyses using model 1 and 2 are presented as least squares (**LS**) means ± standard error (**SE**) for sow hybrid type and parity, F-tests with denominator degrees-of-freedom calculated by Kenward–Roger approximation and corresponding *p*-value. The effect of litter size is represented by the parameter estimate ± SE, *F*-test and *p*-value.

*IUGR.* The effects of sow hybrid and parity on the odds of piglets suffering IUGR were analyzed using mixed effects logistic regression, i.e., a binomial generalized linear mixed effects model (**GLMM**) with logit link.

(Model 3) $$\begin{array}{l} logit\left(\pi_{i}\right)=\beta_{0}+\beta_{1}H_{TN70}+\beta_{2}P_{second}+\beta_{12}H_{TN70}*P_{second}\\ \;+\beta_{3}A_{hut}\;+\beta_{4}F_{low}+\beta_{5}L_{size}+B_{duroc} \end{array}$$

This model included the interaction between hybrid type and parity (*H*_*TN*70_^*^ *P*_*second*_) with a corresponding parameter β_12_. Note that the product will be 1 for the combination of second parity TN70 and 0 otherwise. For the other variables in model 3, please refer to description of model 1 above. Results are presented as odds ratio (**OR**) with 95% confidence interval (**CI**) and *p*-values are from chi-squared tests.

*Litter size, live born and teats.* Birth litter size and number of live born piglets were approximated by a linear mixed effects model as the intended Poisson GLMM could not estimate a random effect of sow. Thus, we are using model 2 but without the fixed effect of litter size.

(Model 4) $$\begin{array}{l} Y_{ijk}=\beta_{0}+\beta_{1}H_{TN70}+\beta_{2}P_{second}\;+\beta_{3}A_{hut}\;+\beta_{4}F_{low}\;\\ \;+\gamma_{j\left(i\right)}+\varepsilon_{ijk} \end{array}$$

The effect of hybrid on the number of teats on the sow was tested with Fisher's exact test due to low counts of the contingency table.

##### Piglet characteristics and risk of death

At outset, the same variables were included as described for characteristics in sow hybrids above. In addition, we included weight at first inspection and IUGR (indicator variable of value 1 if present and 0 otherwise) as fixed effect for analyses of stillbirth and live born deaths before weaning, and rectal temperature at first inspection for analysis of live born deaths. Nevertheless, IUGR was not significant in any of these analyses. The odds of stillborn (model 5) and live born deaths (model 6), respectively, were analyzed with a binomial GLMM and results are presented as OR with 95 %CI and *p*-values from chi-squared tests.

*Stillbirth.* A dichotomous outcome variable was used to describe whether a piglet was diagnosed as stillborn or not (1 = stillborn and 0 = live born). We excluded one litter with extremely many stillborn piglets (seven stillborn and further 2 dying on the day of birth); DanBred of second parity on low protein feed and housed in an A-frame hut, her parturition lasted 35 h. None of these nine piglets were scored as suffering from IUGR and only one had a weight just below 1 kg (960 g) whereas the rest were in the interquartile range of this hybrid and parity combination. This litter obstructed estimation of the random effect of sow. Moreover, the fixed effect of boar hindered convergence and the final model was,

(Model 5) $$\begin{array}{l} logit\left(\pi_{i}\right)=\beta_{0}+\beta_{1}H_{TN70}+\beta_{2}P_{second}+\beta_{3}A_{hut}\;+\beta_{4}F_{low}\\ \;+\beta_{5}W_{D1} \end{array}$$

Here β_5_ is the parameter describing the effect of piglet weight on day 1 *pp, W*_*D*1_.

*Live born deaths.* A dichotomous outcome variable was also used to describe whether a piglet was diagnosed as alive at weaning (0) or not (1, dead between first inspection and 6 weeks *pp*). The analysis included 49 euthanized surplus piglets (43 DanBred and 6 TN70 piglets). Live born death was analyzed using the following final binomial GLMM.

(Model 6) $$\begin{array}{l} logit\left(\pi_{i}\right)=\beta_{0}+\beta_{1}H_{TN70}+\beta_{2}P_{second}+\beta_{3}A_{hut}\;+\beta_{4}F_{low}\\ \;+\beta_{5}W_{D1}+\beta_{6}T_{D1}+B_{duroc} \end{array}$$

In addition to parameters already described above, β_6_ is the parameter for rectal temperature at first inspection, *T*_*D*1_.

## Results

### Exploratory Analyses

Principal component analyses were applied to explore relations among the various recorded variables after standardization. Biplots of the first two dimensions are indicating variables' correlation with the principal components by vectors scaled to fit the plot of individual piglet's value of the components with points that also indicate sow hybrid. Concentration ellipses also indicate hybrid groups by bivariate normal 95% probability contours. These first two dimensions explained 35–38% of the total variance.

**Figure 1:**
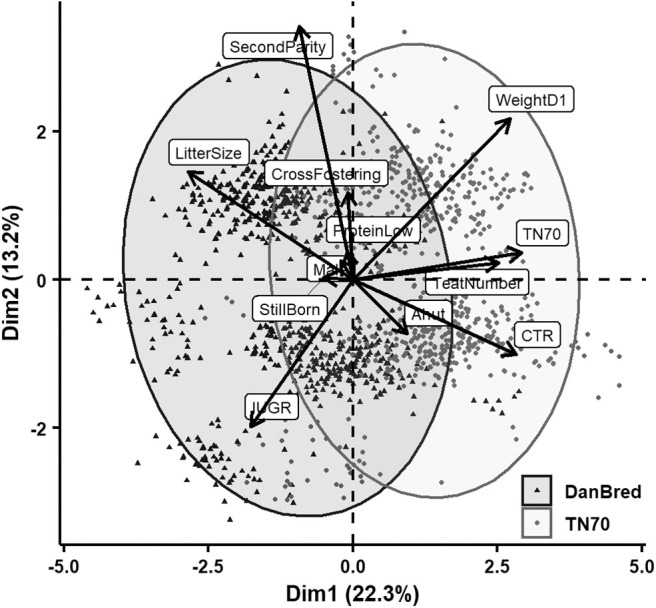
PCA of the relation in the two first dimensions between the recorded variables including both stillborn and live born piglets. Litter size refers to the number of total born piglets in the litter (incl. stillborn), cross fostering refers to whether piglets were cross fostered, second parity to whether the sow gave birth in her second parity (opposite were sows giving birth in first parity), protein low to whether sows received a low protein level in her diet (opposed to normal protein level), male to whether the piglet born was male (opposed to female), stillborn to whether the piglet was stillborn, IUGR to whether the piglet displayed signs of intrauterine growth restriction (opposed to not), Ahut to whether the piglet was born in an A-frame hut (as opposed to a communal hut), CTR to the crown to rump length of the piglet, teat number to the number of teats on the sow, TN70 to whether the piglet was born to a Topigs Norsvin TN70 sow (as opposed to a DanBred sow), and lastly weightD1 to piglet weight recorded on day 1 *postpartum*.

Figure 1 shows the result of the first PCA, which used data from almost all piglets (*N* = 1430) including stillborn (*N* = 42). The largest contribution to the first dimension was attributed to hybrid closely followed by litter size and CTR, both explaining largely the same but obviously in opposite directions. Weight at first presentation also contributed almost equally much to the first dimension whereas number of teats contributed a bit less and almost exactly in the same direction as TN70 hybrid. IUGR was in almost directly opposite direction of weight. Parity had a correlation of 0.85 with the second dimension and contributed most in this direction, followed by IUGR and weight at first presentation. Apart from the clear separation into parities (first parity on the negative and second on the positive part of dimension 2) and hybrid (DanBred on the negative and TN70 of the positive part of dimension 1), DanBred hybrid grouped with larger litter size and IUGR whereas TN70 grouped with higher weight at first presentation, more teats and a larger CTR. The contribution of the other variables is too small to be worth interpreting.

**Figure 2:**
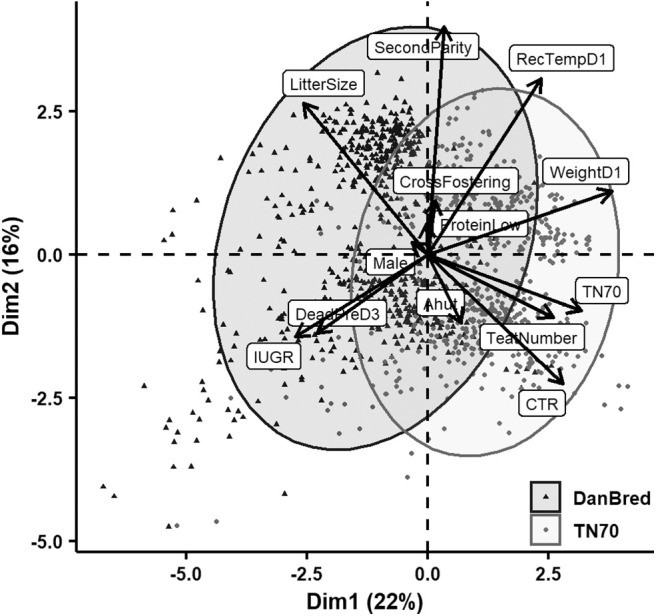
PCA of the relation in the two first dimensions between the recorded variables including live born piglets surviving till at least day 1 *pp*. Litter size refers to the number of total born piglets in the litter (incl. stillborn), cross fostering refers to whether piglets were cross fostered, second parity to whether the sow gave birth in her second parity (opposite were sows giving birth in first parity), protein low to whether sows received a low protein level in her diet (opposed to normal protein level), male to whether the piglet born was male (opposed to female), DeadPreD3 to whether the piglet died before 3 days *postpartum (pp)*, IUGR to whether the piglet displayed signs of intrauterine growth restriction (opposed to not), Ahut to whether the piglet was born in an A-frame hut (as opposed to a communal hut), CTR to the crown to rump length of the piglet, teat number to the number of teats on the sow, TN70 to whether the piglet was born to a Topigs Norsvin TN70 sow (as opposed to a DanBred sow), weightD1 to piglet weight recorded on day 1 *pp*, and RecTempD1 to the rectal temperature of the piglet recorded on day 1 *pp*.

Figure 2 shows the result of the second PCA where all piglets surviving until at least day one were included (*N* = 1296 with full information). In addition to the groupings already mentioned, this indicated grouping of early piglet death (piglets that survived until day 1 but died before day 3 *pp*) with IUGR, low rectal temperature and low weight on day 1 *pp*. Weight had the largest contribution to the first dimension while parity still contributed most to the second dimension followed now by rectal temperature. Weight did not contribute much to the second dimension in this second PCA whereas CTR now contributed more than in the first PCA.

**Figure 3:**
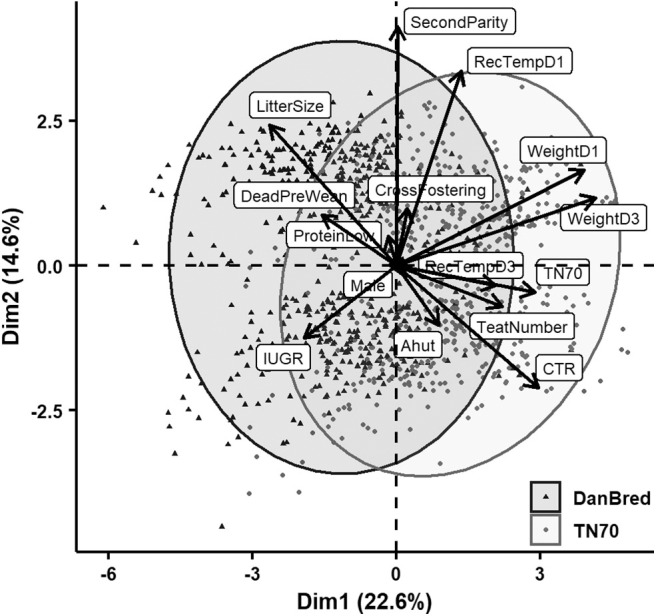
PCA of the relation in the two first dimensions between the recorded variables including live born piglets surviving till at least day 3 *pp*. Litter size refers to the number of total born piglets in the litter (incl. stillborn), cross fostering refers to whether piglets were cross fostered, second parity to whether the sow gave birth in her second parity (opposite were sows giving birth in first parity), protein low to whether sows received a low protein level in her diet (opposed to normal protein level), male to whether the piglet born was male (opposed to female), DeadPreWean to whether the piglet died before weaning (in week 6 *postpartum (pp)* or earlier), IUGR to whether the piglet displayed signs of intrauterine growth restriction (opposed to not), Ahut to whether the piglet was born in an A-frame hut (as opposed to a communal hut), CTR to the crown to rump length of the piglet, teat number to the number of teats on the sow, TN70 to whether the piglet was born to a Topigs Norsvin TN70 sow (as opposed to a DanBred sow), weightD1 to piglet weight recorded on day 1 *pp*, weightD3 to piglet weight recorded on day 3 *pp*, RecTempD1 to the rectal temperature of the piglet recorded on day 1 *pp*, and RecTempD3 to the rectal temperature of the piglet recorded on day 3 *pp*.

Results of the last PCA, where only piglets surviving until at least day 3 *pp* were included (*N* = 1152 with full information), are shown in Figure 3. Late mortality grouped with increasing litter size and the DanBred hybrid. Opposite, the TN70 hybrid grouped with increasing rectal temperature recorded on day 3 *pp*, longer CTR, larger weight (both days) and number of teats on the sow. Weight day 1 and 3 *pp* contributed most and equally to the first dimension followed with half the magnitude by CTR, sow hybrid and litter size. Parity and rectal temperature day 1 *pp* contributed most to the second dimension. Litter size and CTR also contributed some to the second dimension of this PCA and as noted above, these two variables point in opposite directions.

### Piglet Characteristics

**Figure 4:**
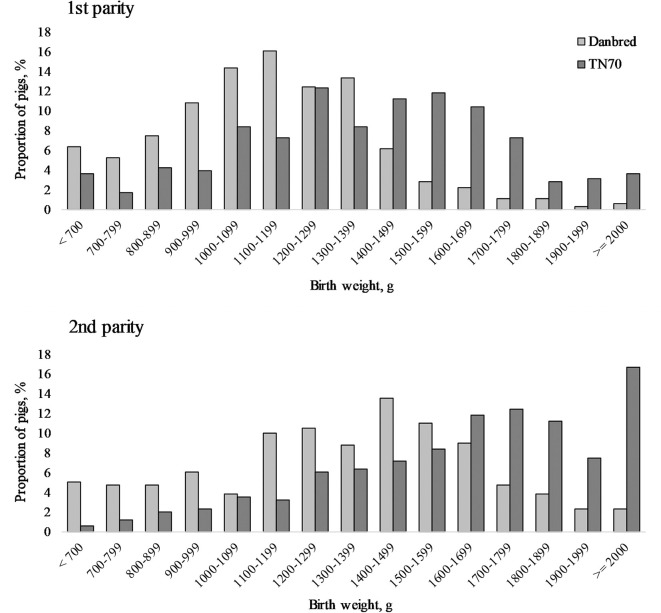
The proportion of piglets in different birth weight intervals of two hybrids [DanBred and TN70 (Topigs Norsvin)] in first and second parity.

In total 1,439 piglets were born in 88 litters during the study period of which 47 litters (22 DanBred and 25 TN70) were born to sows in their first parity while 41 litters (20 DanBred and 21 TN70) were born to sows in their second parity. Across the two parities, DanBred sows gave birth to larger litters and more live born piglets than TN70 sows (Table 1). Litter size (total born) and live born also increased with parity and protein level in the diet, irrespective of sow hybrid. Figure 4 shows the proportion of piglets in different birth weight intervals for the two hybrids and parities.

**Table 1:** Summary of the output from the final models (1 to 4) on piglet characteristics.

**Response**	**Variable**	**Levels**	***LS means±SE***		***F***	***p***
Weight d1 (g, model 1, *N* = 1434)	Sow hybrid	DanBred TN70	1,284 ± 27 1,447 ± 27		*F*_(1, 48.6)_ = 18.6	<0.001
	Parity	1 2	1,206 ± 28 1,525 ± 23		*F*_(1, 47.1)_ = 92.8	<0.001
	Litter size	Continuous			*F*_(1, 72)_ = 93.1	<0.001
Weight d3 (g, model 1, *N* = 1189)	Sow hybrid	DanBred TN70	1,561 ± 32 1,757 ± 31		*F*_(1, 42.3)_ = 20.0	<0.001
	Parity	1 2	1,507 ± 34 1,811 ± 28		*F*_(1, 47.1)_ = 53.8	<0.001
	Litter size	Continuous			*F*_(1, 69.2)_ = 78.2	<0.001
Weight gain (g/day, model 1, *N* = 1014)	Sow hybrid	DanBred TN70	269 ± 6.6 282 ± 6.5		*F*_(1, 40.7)_ = 2.0	0.167
	Parity	1 2	254 ± 8.2 298 ± 6.6		*F*_(1, 58.1)_ = 15.1	<0.001
	Litter size	Continuous			*F*_(1, 79.1)_ = 8.3	0.005
Rec temp 1 (°C, model 2^a^, N=88)	Sow hybrid	DanBred TN 70	38.3 ± 0.09 38.6 ± 0.08		*F*_(1, 40.6)_ =5.6	0.023
	Parity	1 2	38.0 ± 0.08 38.9 ± 0.09		*F*_(1, 41.6)_ =50.0	<0.001
Rec temp 3 (°C, model 2, *N* = 87)	Sow hybrid	DanBred TN70	39.1 ± 0.06 39.2 ± 0.06		*F*_(1, 48.6)_ =1.4	0.249
	Parity	1 2	39.1 ± 0.05 39.2 ± 0.06		*F*_(1, 48.9)_ =1.1	0.301
	Litter size	Continuous			*F*_(1, 79.2)_ =5.7	0.019
	Hut design	Ahut Communal	39.3 ± 0.06 39.1 ± 0.05		*F*_(1, 78)_ =4.1	0.047
Total born (model 4, *N* = 88)	Sow hybrid	DanBred TN70	18.2 ± 0.57 15.7 ± 0.55		*F*_(1, 44)_ =10.3	0.003
	Parity	1 2	15.6 ± 0.53 18.3 ± 0.57		*F*_(1, 44.9)_ =12.7	<0.001
	Protein	Low Normal	16.2 ± 0.54 17.8 ± 0.57		*F*_(1, 67.6)_ =4.7	0.034
Live born (model 4, *N* = 88)	Sow hybrid	DanBred TN70	17.6 ± 0.55 15.4 ± 0.52		*F*_(1, 44.2)_ =8.1	0.007
	Parity	1 2	15.4 ± 0.52 17.6 ± 0.56		*F*_(1, 45.5)_ =8.6	0.005
	Protein	Low Normal	15.7 ± 0.53 17.3 ± 0.55		*F*_(1, 69.5)_ =4.9	0.030
**Response**	**Variable**	**Levels**	***OR***	***95%CI***	***X**^**2**^**(1 df)***	***P***
IUGR (model 3, *N* = 1434)	Hybrid*Parity	DanBred vs. TN70, 1	1.1	0.50;2.2	5.2	0.023
		DanBred vs. TN70, 2	5.1	1.5;17		
		DanBred,1 vs. 2	4.4	1.8;11		
		TN70, 1 vs. 2	21	5.8;77		
	Litter size	Continuous (per 1 extra piglet	1.2	1.1;1.3	10.4	0.001

a *Litter size not included in the final model*.

*First parity sows (parity **1**) had 47 litters and second parity (parity **2**) sows 41 litters. DanBred sows (**DanBred**) had 42 litters and Topigs Norsvin (**TN70**) 46 litters. Weight gain refers to the average daily piglet weight gain in the suckling period and rec temp to piglet rectal temperature on day 1 (d1) and day 3 (d3) postpartum. Total born is the number of piglets born (including both stillborn and live born) and live born is the number of piglets born alive. N refers to the number of used observations. SE is an abbreviation for standard error, OR for odds ratio and 95%CI for 95% confidence interval*.

Sow hybrid and parity affected most of the recorded piglet characteristics (Table 1). TN70 piglets were heavier on both day 1 and 3 *pp* and had a higher rectal temperature on day 1 *pp* when compared to piglets born to DanBred sows. Piglets of second parity sows were heavier on day 1 and 3 *pp*, had a higher average daily weight gain until weaning and had a higher rectal temperature on day 1 *pp* compared to piglets born to first parity sows (Table 1). However, no effects of sow hybrid or parity were found for piglet rectal temperature on day 3 *pp*. Rectal temperature recorded on this day decreased with increasing litter size and was higher for piglets housed in A-frame huts as compared to piglets housed in communal huts.

**Figure 5:**
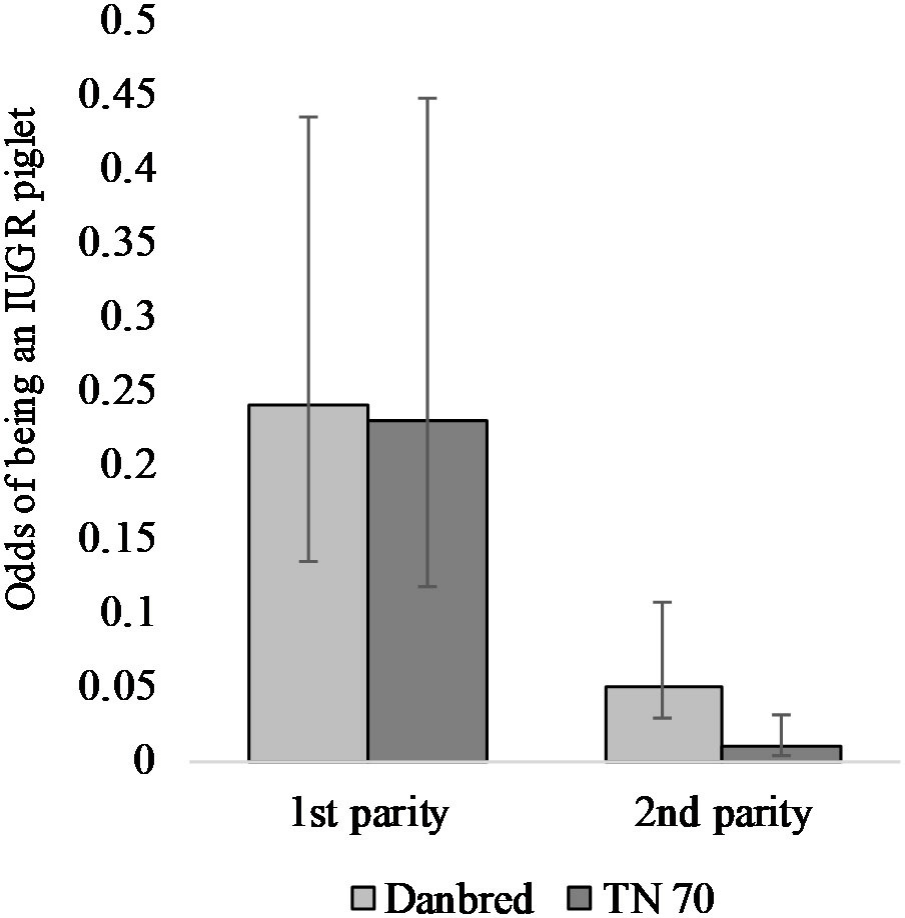
The odds of being an IUGR piglet in two hybrids [DanBred and TN70 (Topigs Norsvin)] for first and second parity. Results are shown with 95% confidence interval bars.

The odds of piglets suffering from IUGR depended on an interaction between sow hybrid and parity (Table 1). The odds of being an IUGR piglet were higher in first compared to second parity for both sow hybrids. Further, the odds were greater in DanBred compared to TN70; this difference was more pronounced in second than in first parity (Figure 5). At first inspection (day 1 *pp*), IUGR piglets had an average weight of 825 ± 210 g (raw mean ± standard deviation (**SD**), 149 piglets) whereas the weight of unaffected piglets was 1436 ± 342 g (1,285 piglets, 5 piglets not included).

### Teat Number

Sow hybrid also influenced the number of teats on the sows (*N* = 49, *p* < 0.001) and TN70 sows had more teats [15, 14–18 teats (raw median, range)] than DanBred sows (14, 12–16).

### Piglet Mortality

On average 0.5 ± 1.0 (raw mean ± SD) piglets per litter were stillborn, 2.8 ± 2.2 of the live born piglets died at 6 weeks of age or earlier (before weaning) and 1.4 ± 1.8 piglet per litter were euthanized either because they were weak (0.8 ± 1.1 piglets per litter, *N* = 72 piglets) or surplus (0.6 ± 1.3 piglets per litter, *N* = 49 piglets). The odds of a piglet being stillborn depended on piglet weight recorded on day 1 *pp* (Table 2) i.e., a 100 g reduction in piglet weight on day 1 *pp* increased the odds of a piglet being stillborn by 30%, corresponding to an odds ratio of 1.3 (95 %CI[1.1;1.4]).

**Table 2:** Summary of the output from the final models 5 (Stillbirth, Included observations = 1414 piglets) and 6 (Live born mortality, Included observations = 1,298 piglets).

**Response**	**Variable**	**Levels**	***OR***	***95%CI***	***X^**2**^ (1 df)***	***P***
Stillbirth (model 5)	Sow hybrid	TN70 vs. DanBred	1.2	0.52; 2.7	0.2	0.697
	Parity	2 vs. 1	3.5	1.6; 7.7	9.3	0.002
	Weight d1 (g)	Continuous (per −100 g)	1.3	1.1; 1.4	19.6	<0.001
Live born death (model 6)	Sow hybrid	Tn70 vs. DanBred	1.0	0.57; 1.8	<0.1	0.993
	Parity	2 vs. 1	3.7	1.9; 7.0	16.0	<0.001
	Weight d1 (g)	Continuous (per −100 g)	1.3	1.2; 1.4	78.8	<0.001
	Rec temp d1 (°C)	Continuous (per −1°C)	2.1	1.6; 2.6	35.6	<0.001

*Weight d1 refers to piglet weight on day 1 postpartum and rec temp d1 to piglet rectal temperature on day 1 postpartum. Results are presented with odds ratio (OR) and 95% confidence interval (95 %CI). Odds ratio is calculated for a 100 g decrease in birth weight and 1°C reduction in rectal temperature*.

No effect of litter size was found for the stillborn odds. The mean total litter size of litters with at least one stillborn piglet was 18.4 ± 4.1 (raw mean ± SD) and 16.1 ± 4.0 for litters without stillborn piglets. The mean piglet weight recorded on day 1 *pp* for the stillborn piglets was 1155 ± 357 g (raw mean ± SD) and 1379 ± 378 g for live born piglets.

In total, 1,397 piglets were live born. Of these 319 died (or were considered non-viable and euthanized) before weaning and 49 needed to be euthanized as surplus piglets. The mean weight and rectal temperature (recorded on day 1 *pp*) of the piglets that died after first inspection (excluding the 49 surplus piglets) were 1280 ± 350 g (raw mean±SD) and 38.1 ± 1.2°C and for piglets that survived until weaning 1467 ± 340 g and 38.6 ± 0.7°C. The weight and rectal temperature of the euthanized surplus piglets were 1013 ± 195g and 38.6 ± 0.7°C, respectively.

The odds of a live born piglet dying were higher when piglets were born to sows in second compared to sows in first parity (Table 2). The odds of live born death also increased when piglets had lower recorded weight and rectal temperature on day 1 *pp* (Table 2).

## Discussion

### Piglet Characteristics in the Two Hybrids

In accordance with the hypothesis, the results showed that sow hybrid affected the characteristics of the piglets born. DanBred piglets were significantly lighter than TN70 piglets at birth and day 3 *pp*. DanBred sows are, among other traits, selected for a high number of live pigs in the litter 5 days *pp* (10) under conventional indoor production conditions with sows in farrowing crates. In contrast the TN70 sows are, among other traits, bred for the sow being able to nurse her own litter (12). DanBred sows were therefore expected to give birth to larger litters than TN70 sows. This was confirmed in the present study where DanBred sows had a larger number of total born piglets than TN70 sows. Larger litters are related to a lower birth weight (15, 18), which likely explains the weight differences found in the present study. A lower birth weight is related to a lower rectal temperature (19), which may explain why piglets born to DanBred sows, in the present study, had a lower rectal temperature on day 1 *pp* compared to TN70 piglets. This relation between piglet weight and rectal temperature may also explain the effect of litter size on rectal temperature recorded on day 3 *pp*. Aside from litter size, rectal temperature on day 3 *pp* also depended on hut design with piglets in A-frame huts having higher recorded temperatures than piglets in communal huts. This difference could be due to the smaller air volume in A-frame huts, which may have made it easier for the sow to warm up the huts than for sows housed in communal huts. Another explanation may be that piglets housed in A-frame huts were processed (weighed etc.) outside, which may have made the observers more alert to the risk of hypothermia (i.e., observers may have been quicker to process each piglet) when compared to piglet processing inside the communal huts.

The odds of a piglet displaying signs of IUGR depended on an interaction between sow hybrid and parity. Both sow hybrids had statistically higher odds of IUGR piglets in their first compared to their second parity. IUGR piglets are commonly lighter than normal/unaffected piglets (20). Accordingly, in the present study, first parity sows gave birth to lighter piglets than second parity sows (despite the lower number of born piglets in first parity) and piglets born to second parity sows had a higher weight gain. This is likely due to the need of first parity sows to allocate resources for their own growth (21) and a lower feed consumption in these compared to in higher parity sows (21, 22). This will pose a greater risk of intrauterine growth retardation particularly in young sows with high prolificacy. The relation between piglet weight and the risk of IUGR (20) could also be part of the reason for the relation between litter size and IUGR in the present study.

Piglet weight and rectal temperature are correlated (19). Hence, the weight differences in early lactation between the two parities likely explain why piglets born to second parity sows had a higher rectal temperature on day 1 *pp* compared to that of piglets born to first parity sows.

### Piglet Mortality

Several of the investigated piglet characteristics affected piglet mortality. The odds of stillborn piglets were higher for lighter piglets and higher when piglets were born to second parity sows. Accordingly, previous studies show a greater risk of stillbirth in piglets with a lighter birth weight both in outdoor (4) and indoor production systems (5, 15, 23). Contrary to previous studies [studies conducted in outdoor production systems (4, 6, 24, 25)] no relation could be shown between litter size and stillbirth risk. This may be because some of the effect of litter size was explained by weight and parity in the present study.

The proportion of stillborn piglets in the present study (raw average 0.5 stillborn piglets/litter) is lower than what was recently found in another Danish study on organic pig production [1.1 stillborn piglets/litter, (25)] and a Danish study on loose-housed indoor sows [1.3 stillborn piglets/litter, (14)]. In both the aforementioned studies, high parity sows were included. The present study only included sows from first and second parity, which may explain the lower proportion of stillborn piglets, as stillbirth increases with increasing parity [e.g., Rangstrup-Christensen et al. (25), Schild et al. (6)]. This was also confirmed in the present study where stillbirth odds was higher for second compared to first parity sows. It may also add to the recorded difference in stillborn rate between the studies that Rangstrup-Christensen et al. (25) relied on stock personnel's visual identification. In the present study, and in the study by Hales et al. (14), *post mortem* examinations were made to determine whether piglets were stillborn or not.

The odds of live born piglets dying before weaning were higher when piglets had a lower weight and a lower rectal temperature on day 1 *pp* and for piglets born to second parity sows. Correspondingly, Hales et al. (14) found higher survivability of piglets with a higher body mass index. Previous studies also show greater risk of mortality in low birth weight piglets (4, 15, 23, 26) and piglets with a lower weight 24 h *pp* (4, 23). A low rectal temperature at 1 h (4, 23, 26) and at 24 h *pp* (4, 23) have also been associated with a greater risk of live born piglets dying. The greater risk of live born death seen for litters born to second parity sows in the present study may be related to the larger litter sizes in second parity.

In the PCA analysis late mortality grouped with DanBred hybrid and increasing litter size. Whilst, the Topigs Norsvin hybrid grouped with increasing rectal temperature recorded on day 3 *pp*, longer crown to rump length, larger weight and number of teats on the sow. However, no effect of sow hybrid was found for the risk of stillborn piglets or live born death until weaning beyond what was explained by differences between hybrids in weight and rectal temperature day 1 *pp*, included as covariates in the model. Thus, the results indicate that the link between DanBred and late mortality found in the PCA is mainly mediated trough litter size affecting birth weight and ability to thermoregulate.

Several studies have shown increased risk of piglet death in piglets displaying signs of IUGR. For example Hales et al. (14) found a higher risk of dying within 1 day *pp* in IUGR piglets compared to normal/unaffected piglets and Amdi et al. (20) related IUGR to e.g., lowered piglet colostrum uptake and glycogen reserves, factors, which may reduce piglet survival chances. It is therefore surprising that no significant relation between IUGR and live born death was found in the present study, although the direction was toward increased odds of death for piglets suffering from IUGR. This may be due to a close relationship between piglet weight and IUGR (20), which is also reflected in all the PCA analyses in the present study (Figures 1–3). When considering the PCA analysis of early live born death, IUGR grouped with death before 3 days *pp*. Late piglet death, (piglets alive at day 3 but dying before weaning, Figure 3), did not group with signs of IUGR on either dimensions. Thus, it is also likely that IUGR is important for early mortality whereas for late mortality other traits are more central, likely due to many IUGR piglets being euthanized before day 3.

### Perspectives

A better match between birth litter size and available teats on the sow is required in organic pig production to reduce the need for nurse sows and the undesirable practice resulting in starvation or euthanasia of surplus piglets, a practice that was performed more often in the hybrid with the highest litter size in the present study. The current level of piglet mortality in organic pig production conflicts with the organic principles of health, which refers to health as “*the maintenance of physical, mental, social and ecological well-being*,” and fairness, which “*insists that animals should be provided with the conditions and opportunities of life that accord with their physiology, natural behavior and well-being*” (7). Furthermore, high mortality constitutes a major welfare concern and lowers the sustainability of organic pig production. Based on the present results it is suggested that use of a less prolific sow hybrid or alternatively, a less prolific sow breed could be beneficial in pig production systems with outdoor farrowing.

Even though both the sow hybrids investigated in the current study were highly prolific, differences in breeding goals have resulted in several differences between the piglets born. Irrespective of litter size, the TN70 sow was accompanied by the birth of piglets displaying traits of better viability. The effects of sow hybrid were mediated through piglet weight and rectal temperature day 1 *pp*.

The study included only young parity sows. Litter size is likely to increase with increasing parity while the rate of increase may depend on hybrid due to different selection goals. In addition to the benefits for piglet viability, use of less prolific sow hybrids will result in the birth of smaller litters. In such litters, there is a better match between the number of piglets born and the number of teats available on the sow, provided the number of teats is not reduced. In spite of their young age, DanBred sows in the present study gave birth to a mean of 18.2 total born piglets and 17.6 live born per litter, despite only having 14.0 (median) available teats. This illustrates the issue that results in surplus piglets. The matter can only be expected to become more pronounced in the later parities where sows give birth to larger litters [parity and birth litter size are related e.g., Koketsu et al. (27), Quesnel et al. (28), Hales et al. (29)] and number of functional teats may decline. TN70 sows had 15.4 available teats, which corresponded to their 15.7 birth litter sizes and 15.4 live born piglets per litter. Whether there continues to be a match between litter size and teats in the TN70 sows in the higher parities needs further investigation. It could be relevant to investigate piglet and sow characteristics of even less prolific sow hybrids (or breeds), which are bred for other traits that could be beneficial in an outdoor farrowing system e.g., thermal tolerance and ability to ingest high amount of feed/roughages to sustain body condition throughout their life. Traits like thermal tolerance (6) and sow body condition (30) have been linked to piglet survival in recent studies on organic outdoor production.

As for other animal production systems, there is a compromise between the requirements for an efficient commercial production system and for achieving higher animal welfare. In a survey conducted prior to the present experiment, Danish organic pig producers were asked to rank qualities that they valued as important with regards to their production animals (31). Among the highest scored qualities were: sows giving birth to a large litter size and maternal ability (e.g., number of functional teats, live born mortality). Since a large litter size is a quality that pig producers value it adds restrictions to the animals, which they may choose to use in their production system. The high scoring of maternal ability, including lower live born mortality, suggests that also producers find the high mortality problematic. Yet, the high priority of large litter sizes raises the question whether it is ethical to continue to pursue breeding goals focussing on this characteristic in outdoor production systems or whether focus should be on traits increasing piglet survival chances.

## Conclusion

The results of the present study showed that sow hybrid affected several of the piglet characteristics, such as piglet weight and rectal temperature, which both related to the risk of piglet death.

Even though both the investigated sow hybrids were highly prolific the differences in breeding goals have resulted in differences in several piglet characteristics related to both pre- and postnatal mortality. Use of less prolific sows giving birth to heavier and fewer piglets in the litter may therefore be a useful tool to reduce piglet mortality in pig production with outdoor farrowing.

## Data Availability Statement

The datasets generated for this study are available on request to the corresponding author.

## Ethics Statement

The animal study was reviewed and approved by Ministry of Environment and Food of Denmark, Danish Veterinary and Food Administration J. nr. 2013–15–2934–00822.

## Author Contributions

S-LS and LP contributed with planning and setting up of the experiment, data collection, editing and analysis. S-LS contributed with writing of the manuscript. LP applied for the project funding and commented on the manuscript. LR-C contributed during data collection and editing, and with commenting the manuscript. LF contributed with data editing and analyses, and with writing of the manuscript. All authors contributed to the article and approved the submitted version.

## Conflict of Interest

The authors declare that the research was conducted in the absence of any commercial or financial relationships that could be construed as a potential conflict of interest.
